# Bax/Tubulin/Epithelial-Mesenchymal Pathways Determine the Efficacy of Silybin Analog HM015k in Colorectal Cancer Cell Growth and Metastasis

**DOI:** 10.3389/fphar.2018.00520

**Published:** 2018-05-23

**Authors:** Haneen Amawi, Noor A. Hussein, Charles R. Ashby, Rawan Alnafisah, Leticia M. Sanglard, Elangovan Manivannan, Chandrabose Karthikeyan, Piyush Trivedi, Kathryn M. Eisenmann, Robert W. Robey, Amit K. Tiwari

**Affiliations:** ^1^Department of Pharmacology and Experimental Therapeutics, College of Pharmacy and Pharmaceutical Sciences, University of Toledo, Toledo, OH, United States; ^2^Pharmaceutical Sciences, College of Pharmacy, St. John's University, Queens, NY, United States; ^3^Biomedical Sciences, College of Veterinary Medicine, Tuskegee University, Tuskegee, AL, United States; ^4^School of Pharmacy, Devi Ahilya Vishwavidyalaya, Indore, India; ^5^School of Pharmaceutical Sciences, Rajiv Gandhi Proudyogiki Vishwavidyalaya, Bhopal, India; ^6^Department of Cancer Biology, University of Toledo, Toledo, OH, United States; ^7^Center for Cancer Research, National Cancer Institute, National Institutes of Health, Bethesda, MD, United States

**Keywords:** silybin, colorectal cancer, tubulin inhibition, metastasis, epithelial-mesenchymal-transition

## Abstract

The inhibition of apoptosis, disruption of cellular microtubule dynamics, and over-activation of the epithelial mesenchymal transition (EMT), are involved in the progression, metastasis, and resistance of colorectal cancer (CRC) to chemotherapy. Therefore, the design of a molecule that can target these pathways could be an effective strategy to reverse CRC progression and metastasis. In this study, twelve novel silybin derivatives, HM015a-HM015k (15a−15k) and compound 17, were screened for cytotoxicity in CRC cell lines. Compounds HM015j and HM015k (15k and 15j) significantly decreased cell proliferation, inhibited colony formation, and produced cell cycle arrest in CRC cells. Furthermore, 15k significantly induced the formation of reactive oxygen species and apoptosis. It induced the cleavage of the intrinsic apoptotic protein (Bax p21) to its more efficacious fragment, p18. Compound 15k also inhibited tubulin expression and disrupted its structure. Compound 15k significantly decreased metastatic LOVO cell migration and invasion. Furthermore, 15k reversed mesenchymal morphology in HCT116 and LOVO cells. Additionally, 15k significantly inhibited the expression of the mesenchymal marker N-cadherin and upregulated the expression of the epithelial marker, E-cadherin. Compound 15k inhibited the expression of key proteins known to induce EMT (i.e., DVL3, β-catenin, c-Myc) and upregulated the anti-metastatic protein, cyclin B1. Overall, *in vitro*, 15k significantly inhibited CRC progression and metastasis by inhibiting apoptosis, tubulin activity and the EMT pathways. Overall, these data suggest that compound 15k should be tested *in vivo* in a CRC animal model for further development.

## Background

Colorectal cancer is the second leading cause of cancer death in the United States and the third leading cause of cancer death worldwide. In 2016, the American Cancer Society predicted the diagnosis of 134,490 new cases of colorectal cancer (American-Cancer-Society, 2016). Although the 5-year survival rate for localized CRC is >90%, only 39% of all cases are detected at this largely asymptomatic stage. Furthermore, advanced metastatic disease is usually resistant to treatment, with survival rates of only 12% (American-Cancer-Society, 2016). Approximately 5–10% of CRC cases are due to familial adenomatous polyposis, which is caused by inherited mutations in the tumor suppressor gene *APC* (about 1% of all CRC cases) (Half et al., 2009). Non-familial CRCs are more common (≈ two third of the cases) and are frequently associated with alterations in several molecular pathways, including over-activation of the epidermal growth factor receptor (EGFR) (Markman et al., 2010; Yarom and Jonker, 2011), alterations in the embryonic development pathways (Wnt/β-catenin-EMT) (Bates and Mercurio, 2005; Bertrand et al., 2012), inhibition of apoptotic signaling pathways (Bedi et al., 1995; Watson, 2004; Zhang and Yu, 2013), and dysregulation of microtubule dynamics (Carles et al., 1999; Giarnieri et al., 2005; Zhao et al., 2016).

The currently available antineoplastic medications that increase patient survival include conventional cytotoxic drugs as well as “targeted” therapeutics (Aparo and Goel, 2012; Gonzalo et al., 2014). However, these aforementioned treatment regimens are limited as they elicit severe adverse effects and toxicities (Alagoz et al., 2012; Gilbert et al., 2012). In addition, the development of resistance to these drugs is a common problem that results in chemotherapy failure (Polyak and Weinberg, 2009; Tiwari et al., 2011; Zhang and Guo, 2016). Consequently, there is an essential need to develop and design new therapeutic drugs with significant anticancer efficacy, limited toxicity, and most importantly, efficacy against resistant metastatic colorectal cancer.

The role of epithelial to mesenchymal transition (EMT) in the development of cancer progression and metastasis is well-established (Cao et al., 2015; Amawi et al., 2017a). Several EMT—related signaling pathways and proteins have been reported to mediate the development of CRC metastasis and resistance (Brabletz et al., 2005). Accordingly, targeting EMT and its associated proteins represents a novel approach to reverse CRC metastasis and resistance (Du and Shim, 2016).

**Figure 1:**
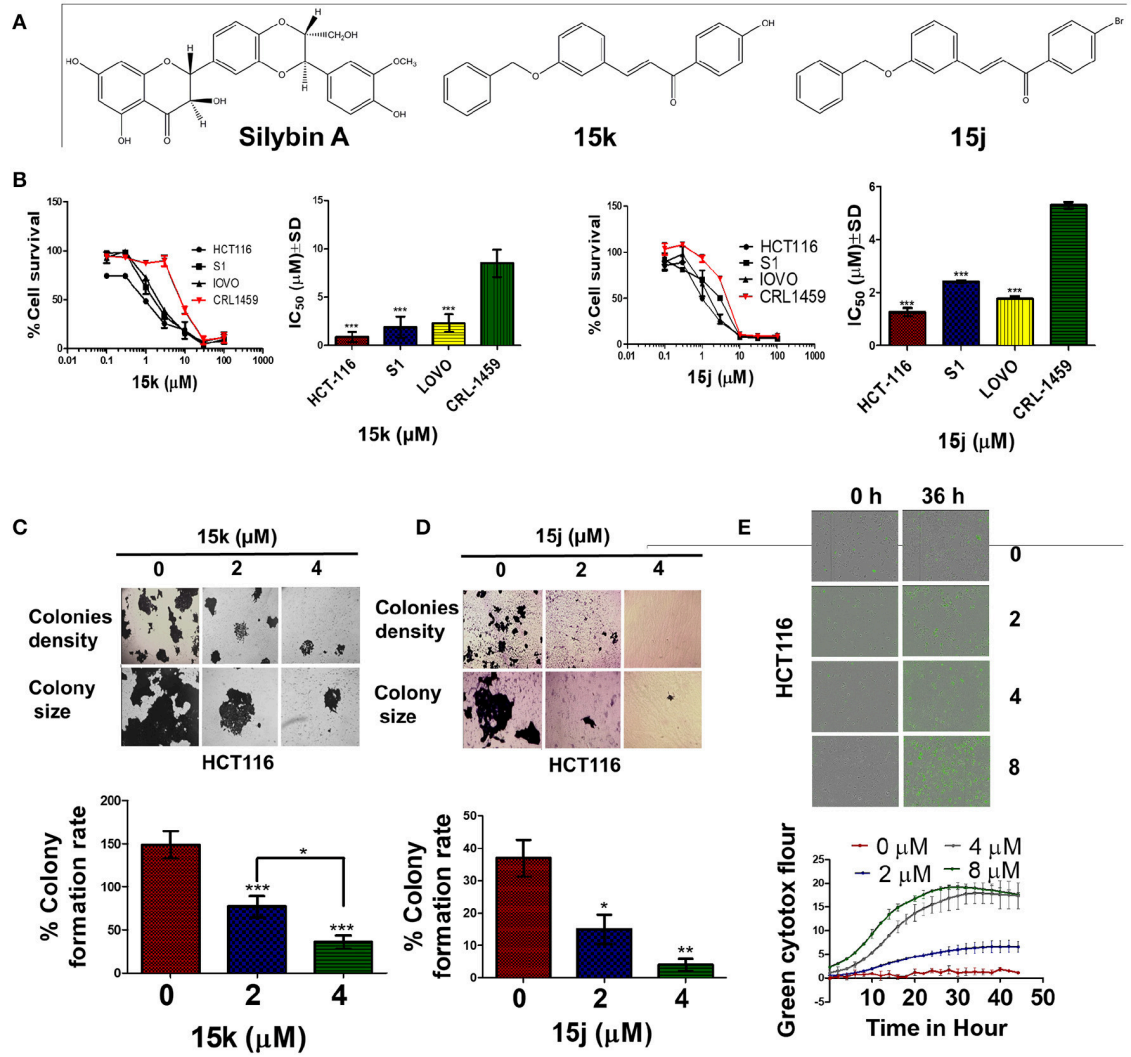
The selectivity and cytotoxicity of 15k, 15j on colon cancer cell lines; **(A)** The chemical structures of silybin A and the two potential lead silybin derivatives 15k, 15j; **(B)** Survival of colon cancer cells (HCT116, S1, LOVO) compared to that of normal colon cells (CRL1459); IC_50_ Values of 15k, 15j respectively on colon cancer cells (HCT116, S1, LOVO) compared to that of normal colon cells (CRL1459); Cell survival was determined by the MTT assay. IC_50_ values are represented as means ± SD of three independent experiments performed in triplicate. Statistically, ^***^P < 0.001; **(C,D)** Colony formation assay with quantification of colony number represented as colony formation rate. HCT116 CRC cancer cells were incubated with different concentrations (0, 2, 4 μM) of 15k and15j. The pictures show the effect of 15k **(C)**, and 15j **(D)** on colony formation in whole well, colonies density, and colony size; a bar graph summarizing the results for 15k and 15j, respectively. The results are represented as means ± SD of three independent experiments with ^*^p < 0.05, ^**^p < 0.01, ^***^p < 0.001. **(E)** Green cytotox (green fluorescence) to quantify cell proliferation and death; Representative pictures of the fluorescence level green cytotox at the 0 and 36 h time points; time line curve quantitatively summarizing the results is also shown. The data are presented as the means ± SEM of three independent studies.

We previously reported the design and synthetic schemes for 12 novel silybin derivatives. The derivatives were found to be efficacious and selective for ovarian cancer cell lines OV2008 and A2780 (Figure 1A, silybin structure) (Manivannan et al., 2017). However, their pharmacodynamics mechanisms remained to be elucidated. Therefore, in this study, the compounds were tested in CRC cell lines and compared to normal, non-cancerous cell lines to determine their potential efficacy and selectivity. In addition, detailed experiments with the lead compound, 15k (structure, Figure 1A), were conducted to determine its efficacy to (1) induce cell cycle arrest; (2) induce reactive oxygen species; (3) activate apoptosis, mainly through cleavage of the proapototic protein Bax, and subsequent caspase 3 activation; (4) inhibit tubulin protein expression and activity; and (5) reverse epithelial-mesenchymal transition (EMT).

## Methods

### Reagents

The α- tubulin, β- catenin, β- actin, Bax, Bak, Bcl-2, caspase 3, E-cadherin, N-cadherin, c-Myc, cyclin B1, and histone antibodies were purchased from Cell Signaling Technology (Danvers, MA, USA). Mitochondrial membrane potential/annexin V apoptosis kit and propidium iodide dyes were purchased from Life Technologies (Eugene, Oregon, USA). Dulbecco's modified Eagle's medium (DMEM) was purchased from GE Healthcare Life Sciences, HyClone Laboratories (Logan, Utah, USA). Fetal bovine serum was purchased from Atlanta biologicals (Flowery Branch, GA, USA). 3-(4,5-dimethylthiazol-2-yl)-2,5-diphenyltetrazolium bromide (MTT) was purchased from Calbiochem EMD Millipore (Billerica, MA, USA). 2′,7′-Dichlorofluorescin diacetate powder was purchased from Sigma Aldrich (St. Louis, MO, USA 63146). Bicinchoninic acid (BCA) solution and copper solution were purchased from G-Biosciences (St. Louis, MO, USA). 0.25% trypsin + 2.2 mM ethylenediaminetraacetic acid (EDTA) and phosphate buffer saline (PBS) were purchased from Mediatech, Inc. (Corning Subsidiaries, Manassas, VA, USA). Paraformaldehyde, 500 mg (powder form), was purchased from Fisher Scientific (Hampton, NH, USA). Mini-Protean® TGX™ precast Gels, Clarity™, and Clarity Max™ Western ECL Blotting Substrates were purchased from BIO-RAD Laboratories (Hercules, CA, USA). Polyvinylidene difluoride (PVDF) membranes were purchased from Thermo Fisher Scientific (Waltham, MA, USA). Non-fat dry milk was purchased from Cell Signaling (Danvers, MA, USA). Tween-20 was purchased from Fisher Scientific (Springfield Township, NJ, USA).

### Cell lines and culture

Colorectal cancer cell lines, including HCT116, LOVO, and S1, with varying drug sensitivity and invasiveness, were grown as adherent monolayer in culturing flasks. Normal epithelial cells lines (Chinese hamster ovarian: CHO, normal epithelial colon: CRL1459) were also grown. Both cancerous and normal cell lines were kindly obtained from the late Dr. Gary Kruh (University of Chicago, Illinois). In addition, CRC HCT116 Bax knockout (Bax–/–) cells were kindly provided by Dr. Bert Vogelstein (Johns Hopkins, Baltimore) (Zhang et al., 2000), Bak knockout (Bak–/–) and Bax-Bak double knockout (Bax–/– and Bak–/–) cells were kindly provided by Richard Youle (NIH, Baltimore, MD) (Wang and Youle, 2012). DMEM, with 4.5 g of glucose, was supplemented with 10% fetal bovine serum (FBS) and 1% penicillin/streptomycin and used for all cell lines. The cells were cultured in a humidified incubator containing 5% CO_2_ at 37°C. All of cells were checked and confirmed to be free of fungi and mycoplasma. Cells were obtained from frozen stocks and cell passaging (up to P4) was performed at 80% cell confluency by PBS and trypsin + 2.2 mM EDTA.

### Cell cytotoxicity assays

#### MTT assay

The 3-(4,5-dimethylthiazol-2-yl)-2,5-diphenyltetrazolium bromide (MTT) assay was used to determine the sensitivity of the different CRC cells to the novel silybin derivatives (Hussein et al., 2017; Manivannan et al., 2017). The cells were seeded at a density of 4,000–5,000 cells/well in 96 well plates and incubated with serial dilutions of the compounds (0, 0.1, 0.3, 1, 3, 10, 30, and 100 μM). The MTT dye (4 mg/ml) was added after 72 h of incubation and incubated with the cells for an additional 4 h at 37°C. Following incubation, the media was discarded and the formazan crystals were dissolved by adding 100 μL of DMSO to each well. A DTX 880 multimode detector (Beckman Coulter Life, Indianapolis IN, USA) was used to determine the absorbance readings at a wavelength of 570 nm. The IC_50_ values were determined based on 3 separate experiments, with each experiment done in triplicate. The selectivity of the compounds was determined by comparing their cytotoxicity for CRC to normal epithelial cell lines (CHO, and CRL1459).

#### Colony formation assay

In this assay, HCT116 cells were seeded in 6 well plates at a density of 250,000 cells/plate and allowed to grow overnight. Compounds 15j and 15k (0, 2 or 4 μM) were added the following day. After 12 h of incubation, the medium was discarded and cells were trypsinized with 0.25% trypsin, 2.21 mM EDTA, 1X, harvested, counted and reseeded in 6 well plates at a low density (500 cell/well). The cells were allowed to form colonies for 10–14 days at 37°C, with the medium being changed every other day. Subsequently, methanol was used to fix the colonies formed in each plate, followed by dyeing the colonies with 0.1% crystal violet dye for 30 min. Finally, colonies were viewed and counted under an EVOS microscope (Themo Fisher Scientific, Wayne, MI, USA). The colony formation rate equation was applied for each compound: Colony formation rate = number of colonies/number of seeded cells × 100 %.

#### IncuCyte™ cytotox green reagent to detect dead cells

For real time quantification of cell death, the Incucyte Cytotox green reagent was used. It is a highly sensitive cyanine nucleic acid dye that when added to normal healthy cells, it does not significantly affect cell growth or morphology and yields little or no intrinsic fluorescent signal. The plasma membrane integrity is decreased in unhealthy cells, allowing entry of the IncuCyte Cytotox reagent, yielding a 100- to 1,000-fold increase in fluorescence upon binding to deoxyribonucleic acid (DNA) (at an excitation maximum of 491 nm and an emission maximum of 509 nm). The HCT116 cells were seeded at a density of 1,000 cell/well and allowed to grow overnight. The next day, compound 15k at 0, 2, 4, and 8 mM was prepared and added, directly followed by the Cytotox green reagent, to the cells, at a final concentration of 0.25 μM. The cells were then incubated in a IncuCyte Zoom live cell imaging apparatus and pictures were taken every 2 h for up to 50 h.The IncuCyte® integrated analysis software was used to quantify the fluorescent objects and minimize the background fluorescence (IncuCyte ZOOM version 2016A, Essen BioScience, Ann Arbor, MI 48108, USA).

### Cell cycle analysis

Cell cycle analysis was performed as described previously (Pozarowski and Darzynkiewicz, 2004). Briefly, a 6 well plate was used for the seeding of HCT116 at 1 × 10^6^ cells/well. The next day, 15j and 15k (0, 2, or 4 μM) were added and incubated with the seeded cells. After 12 h, the cells were trypsinzed with 0.25% trypsin, 2.21 mM EDTA, 1X, washed, counted and resuspended in 0.5 ml of ice cold PBS. PI stain was added to each suspension for at least 15 min to stain the DNA. The cell cycle analysis and cell distribution for each phase was measured using a BD Accuri™ C6 flow cytometer (BD Biosciences, Becton-Dickinson, San Jose, CA, USA) and analyzed using FCS express 5 plus De Novo software (Glendale, CA, USA).

### Detection of reactive oxygen species (ROS)

H_2_DCF-DA was used as previously described (Amawi et al., 2017c). The cells that had been incubated with the test compound were further incubated with 3 μM of the H_2_DCF-DA working solution for 30 min at 37°C. The fluorescence level of oxidized DCF (excitation at 485 nm and emission at 535 nm) was detected under EVOS digital fluorescent microscope at 40x.

### Apoptosis assays

#### Hoechst 33258 staining

Nuclear condensation was detected using Hoechst 33258 DNA dye. HCT116 cells were seeded at 1 × 10^5^ cells/well in 6-well plates and incubated overnight at 37°C. The following day, cells were exposed to 15k or 15j (0, 2, and 4 μM) and further incubated overnight at 37°C. The cells were fixed and stained with Hoechst 33258 for at least 30 min. An EVOS digital microscope was used to detect the stained nucleus fluorescence at wavelengths of 460–490 nm.

#### Determination of mitochondrial membrane potential and induction of apoptosis

The mitochondrial membrane potential and the induction of apoptosis were analyzed in HCT116 cells using the MitoTracker Red and Alexa Fluor 488 annexin V kits for flow cytometry as previously described (Karthikeyan et al., 2015). Six well plates were used for seeding of cells, followed by incubation with 15j or 15k (0, 1, 2, and 4μM) for overnight. Subsequently, the cells were harvested, counted and 4 μl of a 10μM working solution of MitoTracker Red was added to each 1 ml cell suspension, followed by 30 min of incubation at 37°C with 5% CO_2_. The cells were resuspended in 100 μl of annexin binding buffer and 5 μl of Alexa Fluor 488 annexin V was added to the suspension and incubated for 15 min. Finally, 400 μl of annexin-binding buffer was added and the stained cells were detected by flow cytometry. The fluorescence emission at 530 and 585 nm was measured using a BD Accuri™ C6 flow cytometer and analyzed using FCS express 5 plus De Novo software.

#### IncuCyte™ caspase-3/7 and annexin V red reagents for apoptosis

For real time quantification of apoptosis induction, the fluorescent Caspase 3/7 and Annexin V red reagents, with an excitation maximum of 500 and 593 nm and an emission maximum of 530 and 614 nm, respectively, were used. The HCT116 cells were seeded at a density of 1,000 cell/well and allowed to grow overnight. The next day, compound 15k at 0, 2, 4, and 8μM was prepared and added, directly followed by the caspase 3/7 reagent at final concentration of 5μM or annexin V red reagent in final dilution of 1: 200. The cells were then incubated in IncuCyte Zoom live cell imaging and pictures were taken every 2 h for up to 50 h and analyzed using the integrated software. The IncuCyte® Caspase-3/7 apoptosis assay reagent couples the activated caspase-3/7 recognition motif (DEVD) to NucView™ 488, a DNA intercalating dye to enable quantification of apoptosis over time. Caspase-3/7 assay reagent is non-perturbing to cell growth and morphology. When added to cell culture medium, this inert, non-fluorescent substrate crosses the cell membrane, where it is cleaved by activated caspase-3/7, resulting in the release of the DNA dye and green fluorescent staining of nuclear DNA.

### Migration and invasiveness assays

#### Wound healing assay

In brief, the metastatic CRC cells (LOVO) were seeded in 6 well plates and allowed to grow as a monolayer until they reached 100% confluence. Upon confluence, 200 μl sterile tips were used to create a wound by gentle scratching of the complete cell monolayer. Sterile PBS was used to wash the cells several times to remove any floating cells. Different concentrations of the test compound were prepared in the culture media and added immediately after wound formation. The closure of the wound was observed by taking pictures at different time points by an EVOS microscope. Finally, the area of the wounds at different time points was calculated using Image J software (NIH, Bethesda, Maryland, USA).

#### Transwell migration assay

The effect of compound 15k on the invasiveness of LOVO was tested using 24 trans-well inserts with an 8μM pore size. The addition of the insert to the well forms two chambers (upper and lower), where the upper chamber was used to grow the cells on the porous membrane. The lower chamber was filled with 600 μl of cell-free DMEM medium. A 200 μl of cell suspension was added to each insert and allowed to attach for 1 h. The test compound was added in different concentrations and incubated with the cells for 24 h. Cells that did not migrate were removed from the upper chamber by a cotton swab. Finally, the remaining migrated cells were fixed by methanol and dyed with 0.1% crystal violet dye and observed with an EVOS microscope. The number of migrated cells was counted and compared between the treated and control cells.

### Subcellular fractionation and western blot

HCT116 cells were lysed to obtain cytoplasmic and nuclear protein fractions, according to the protocol used in previous publications (Alhadidi and Shah, 2017). Briefly, the cells were seeded in and incubated with 15k for overnight at 4μM. The cells were washed using ice-cold PBS and scraped using a cell scraper and collected in 15ml tubes. The cells were centrifuged and the PBS was discarded and replaced with 400 μl of buffer A (10 mM HEPES, pH 7.9, 0.1 mM EDTA, 0.1 mM EGTA, 10 mM KCl, 1 mM dithiothreitol, and 0.5 mM phenylmethylsulfonyl fluoride) in each tube. The cells were allowed to swell under in the buffer for 15 min on ice. Subsequently, 0.1% Nonidet P-40 was added to each suspension for 1 min to disturb the plasma membrane. The nuclei were precipitated by centrifugation at 13,000 rpm for 1 min at 4°C. The cytoplasmic proteins were collected in the supernatant and stored at −80°C. Two hundred μl of Buffer B (20 mM HEPES, pH 7.9, 1 mM EDTA, 1 mM EGTA, 400 mM NaCl, 1 mM dithiothreitol, and 1 mM phenylmethylsulfonyl fluoride) was added to the pellets and left for 30 min on ice with periodic mixing. The samples were centrifuged at 13,000 rpm for 15 min at 4°C to obtain the nuclear fraction (supernatant). The bicinchoninic acid (BCA) quantification assay was used to determine the concentration of protein in the cell extracts. The Western blot was done as previously described (Tiwari et al., 2013). Rabbit α-tubulin, 1:4,000 dilution, rabbit Bax (1:1,000), rabbit BAK (1:1,000), rabbit β-Catenin (1:4,000), rabbit DVL3 (1:4,000), mouse β-actin (1:5,000), E-cadherin (1:4,000), and N-cadherin (1:4,000), antibodies were used. Membranes were then washed and incubated for 1 h with horseradish peroxidase-labeled (HRP) anti-rabbit and anti-mouse secondary antibodies (1:5,000 dilutions). ChemiDoc Imaging Systems from Bio-Rad, (Hercules, California USA) was used to detect the blots. Finally, proteins were quantified using the image J software. Data were calculated as ratios to β-actin.

### Immunofluorescence staining

HCT116 cells were used to ascertain the action of compound 15k on α-tubulin, E-cadherin and β-catenin. The cells were seeded on to culture glass covers inserted in 6 well plates. The compound was added at 0, 2, and 4μM and incubated with the cells overnight at 37°C. Paraformaldehyde (4%) was used for cell fixation, followed by permiabilization with 0.3% triton 100× in PBS for 25 min. The cells were blocked with 3% BSA in PBS for 30 min. The α-tubulin, E-cadherin, or β-catenin rabbit antibodies in the correct dilution were added, followed by fluorescent anti-rabbit secondary antibody in the same manner. The nuclei were stained with the nuclear dye, DAPI, for 15 min. The EVOS cell imaging system was used to detect the fluorescence from each slide.

### Statistical analysis

A two-way ANOVA, followed by Bonferroni *post-hoc* analysis, was used for data obtained from the wound healing assay, cell cycle assay, MitoTracker Red and Alexa Fluor 488 annexin V assay for apoptosis and analysis of β-catenin Western blots. A one-way ANOVA, with Tukey *post-hoc* analysis, was used for the data obtained from the colony formation assay, Western blots of the knockout cells, transwell migration assay, Hoechst staining, immunofluorescence and IC_50_ comparison of 15k in the different HCT116 knockout models. Finally, Student's *t*-test was used to analyze all other Western blot data. All of the experiments were repeated in triplicate. The results were expressed as the mean ± the standard deviation (SD). The *a priori* significance level was *p* < 0.05.

## Results

### Structure-activity relationship of new silybin analogs in CRC cells

A series of diverse silybin analogs were tested for their cytotoxic potential in three human CRC cells HCT-116, S1, LOVO, and two normal cell lines CRL1459 and CHO, using the MTT assay. The cytotoxic activity data of the compounds are presented as IC_50_ values in Table 1.

**Table 1:** The effects of the silybin derivative compounds on the survival of CRC cell lines and normal cell lines.

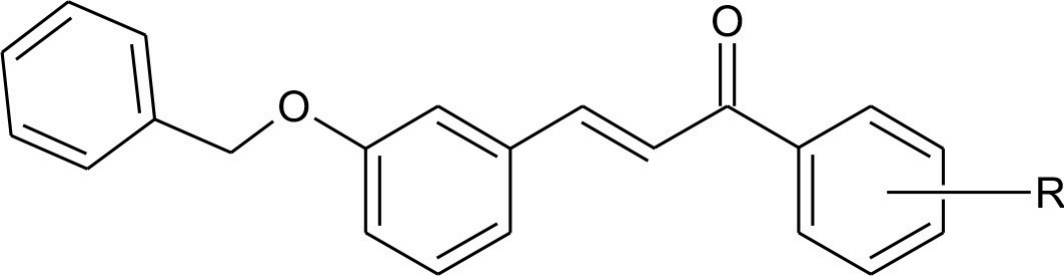						
**Comp. Code**	***R***	**IC**_**50**_ ± **SD (**μ**M)**				
		**Colon**			**Normal**	
		**HCT116**	**LOVO**	**S1**	**CRL-1459**	**CHO**
15a	H	6.4 ± 0.2	11.5 ± 0.8	2.8 ± 0.5	7.8 ± 1.0	22.0 ± 1.7
15b	4-Cl	12.7 ± 1.4	36.5 ± 1.5	9.5 ± 1.4	12.8 ± 2.1	43.6 ± 1.9
15c	2,4-di Cl	9.0 ± 1.1	6.7 ± 2.2	6.9 ± 1.1	7.8 ± 1.1	27.5 ± 1.2
15d	4-NO_2_	22.7 ± 4.2	36.3 ± 9.1	9.0 ± 1.2	26.0 ± 1.6	82.4 ± 1.7
15e	4-CH_3_	16.5 ± 3.1	36.5 ± 1.3	22.4 ± 1.9	19.8 ± 1.3	26.4 ± 0.9
15f	4-OCH_3_	44.7 ± 3.1	11.8 ± 3.5	23.4 ± 2.4	25.4 ± 1.9	22.9 ± 0.3
17		7.3 ± 2.4	8.9 ± 2.1	2.6 ± 0.6	6.4 ± 0.4	23.0 ± 1.3
15g	2,3,4-tri Cl	0.4 ± 0.0	2.4 ± 0.4	2.3 ± 0.7	2.5 ± 0.2	8.6 ± 0.9
15h	2-Br	0.7 ± 0.2	1.9 ± 0.3	1.9 ± 0.5	2.4 ± 0.9	9.1 ± 0.2
15i	3-Br	1.9 ± 0.1	7.5 ± 0.9	6.2 ± 1.9	7.2 ± 0.9	9.2 ± 0.4
15j	4-Br	1.0 ± 0.2	1.8 ± 0.4	2.4 ± 0.7	5.4 ± 1.0	9.0 ± 0.5
15k	4-OH	0.9 ± 0.0	2.3 ± 0.2	1.9 ± 0.9	8.5 ± 0.7	8.1 ± 1.2

*Cell survival was determined by MTT assay as described in section Materials and Methods. The IC_50_ values are represented as mean ± SD of three independent experiments performed in triplicate. The compounds were screened on CRC cell lines (HCT116, LOVO, and S1) and normal cell lines (CRL-1459, CHO)*.

As evident from the table, the silybin analogs (15a–15k) were efficacious in CRC cells, with IC_50_s ranging from 0.4 to 44.7 μM in the CRC cells and 2.4–82.4 μM in the normal cancer cells. Interestingly, the tested compounds display a differential magnitude of selectivity index between cancer cells and normal cells. The halogenated silybin analogs, specifically the bromo- substituted (15h–15j) and 4-hydroxyl- substituted (15k) silybin analogs, were the most efficacious among the series, with several compounds showing submicromolar to <5μM potency in the three CRC cells. For the bromo substituted analogs (15h–15j), the positioning of the bromo group appears to significantly affect the cytotoxic and the ortho substitution yields the most efficacious compound (15h), followed by the compounds with the para (15j) and meta substitutions (15i). Similarly, compound 15g, which also has ortho halogen substitutions, (2,3,4-tri chloro) was 22-fold more than the 3,4-dichloro substituted compound, 15c, and three-fold more efficacious in LOVO and S1 cells. Compound 15k, with a hydrophilic 4-hydroxyl substituent, had a similar cytotoxic profile to that of the brominated analogs, with IC_50_ values of 0.9, 2.3, and 1.9 μM in HCT-116, LOVO, and S1 cells, respectively. Because compounds 15k and 15j (Figure 1A) were selectively cytotoxic in CRC cells compared to normal cells, we conducted additional experiments to determine their cytotoxic mechanism(s) (Figure 1B).

### 15j and 15k have selective cytotoxicity, while inducing cell cycle arrest at different phases in CRC cells

Consistent with the MTT cytotoxicity data, compound 15k significantly inhibited HCT116 colony formation in a concentration—dependent manner (Figure 1C). The mean colony formation rate was decreased significantly in cells incubated with 2 and 4 μM, respectively, of compound 15k, compared to the vehicle control cells (*p* < 0.001, Figure 1C). Similarly, compound 15j produced a significant concentration-dependent decrease in the colony formation rate (Figure 1D), where 2 and 4 μM of compound 15j significantly decreased colony formation rate compared to control (*p* < 0.05 and *p* < 0.01, respectively). Furthermore, the compounds also significantly decreased the size of the colonies formed, as seen in Figure 1D. Subsequently, we conducted experiments to determine if compound 15k produced a time-dependent effect on cell death. As shown in Figure 1E, the cytotoxicity of compound 15k was determined in HCT116 cells over a time period of up to 45 h at different concentrations (0, 2, 4, and 8 μM). The HCT116 cells had intense fluorescence over time compared to the cells incubated with vehicle, with a significant difference in the number of dead cells after 24 h of incubation with 2 μM of 15k. However, the 4 and 8 μM concentrations induced a significant increase in cell death compared to control cells at earlier time points (12 h; Figure 1E). The cytotoxicity of 15k and 15j in HCT116 cells were further determined using a more homogeneous CellTiter-Glo® luminescent cell based assay that measures the ATP content in the metabolically active cells. The ATP depletion produced by 15k and 15j resulted in IC_50_ values (0.76 and 0.69 μM, respectively) similar to those obtained in MTT assays (Data not shown).

In order to gain insight into the mechanism by which the sylibin derivatives 15j and 15k inhibit HCT116 CRC cells proliferation, we conducted experiments to determine their effect on the cell cycle. Compound 15k produced a significant concentration-dependent increase in the number of cells in the G2 and S phases, compared to control (Data not shown). In contrast, compared to 15k, compound 15j significantly increased the level of cells in the sub G1 phase in a concentration—dependent manner and inhibits HCT116 cell growth and proliferation by blocking cell cycle at the sub G1 phase (data not shown).

### 15k induces reactive oxygen species formation in CRC cells

**Figure 2:**
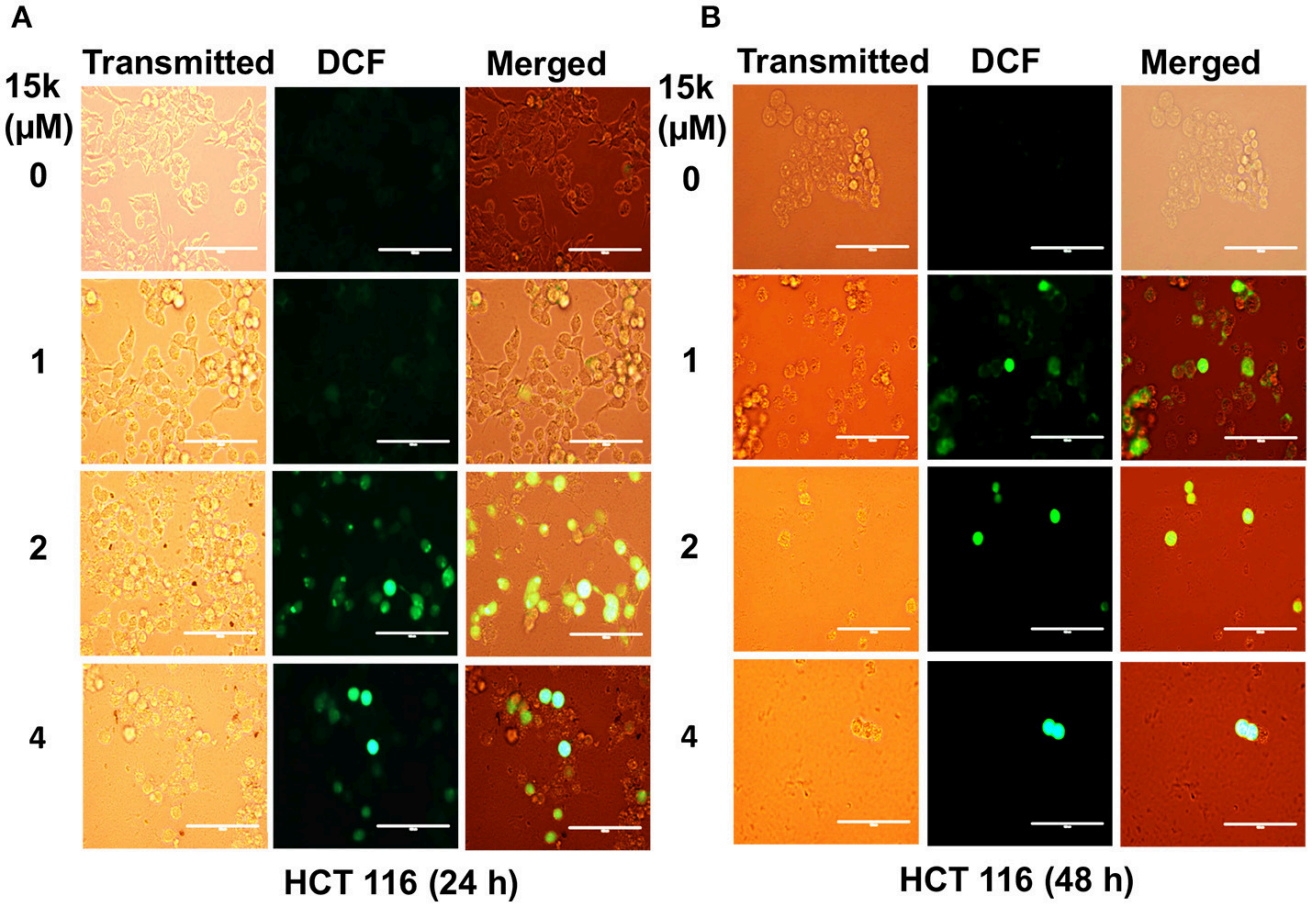
The effect of 15k on the generation of excessive oxidative stress in HCT116 cells; **(A,B)** Representative images of fluorescent DCF levels following the incubation with 1, 2, and 4 μM of 15k for 24 and 48 h, respectively. Representative images from three independent experiments are shown.

The levels of fluorescence of oxidized DCF were measured as an indication of the level of cellular oxidative stress. 15k (1, 2, and 4 μM) significantly increased ROS formation in HCT116 cells compared to vehicle-incubated cells (Figure 2), as indicated by higher green fluorescence (*p* < 0.01 for 2 and 4 μM at 24 and 48 h, Figures 2A,B, respectively).

### Compounds 15k and 15j significantly induce apoptosis in CRC cells

**Figure 3:**
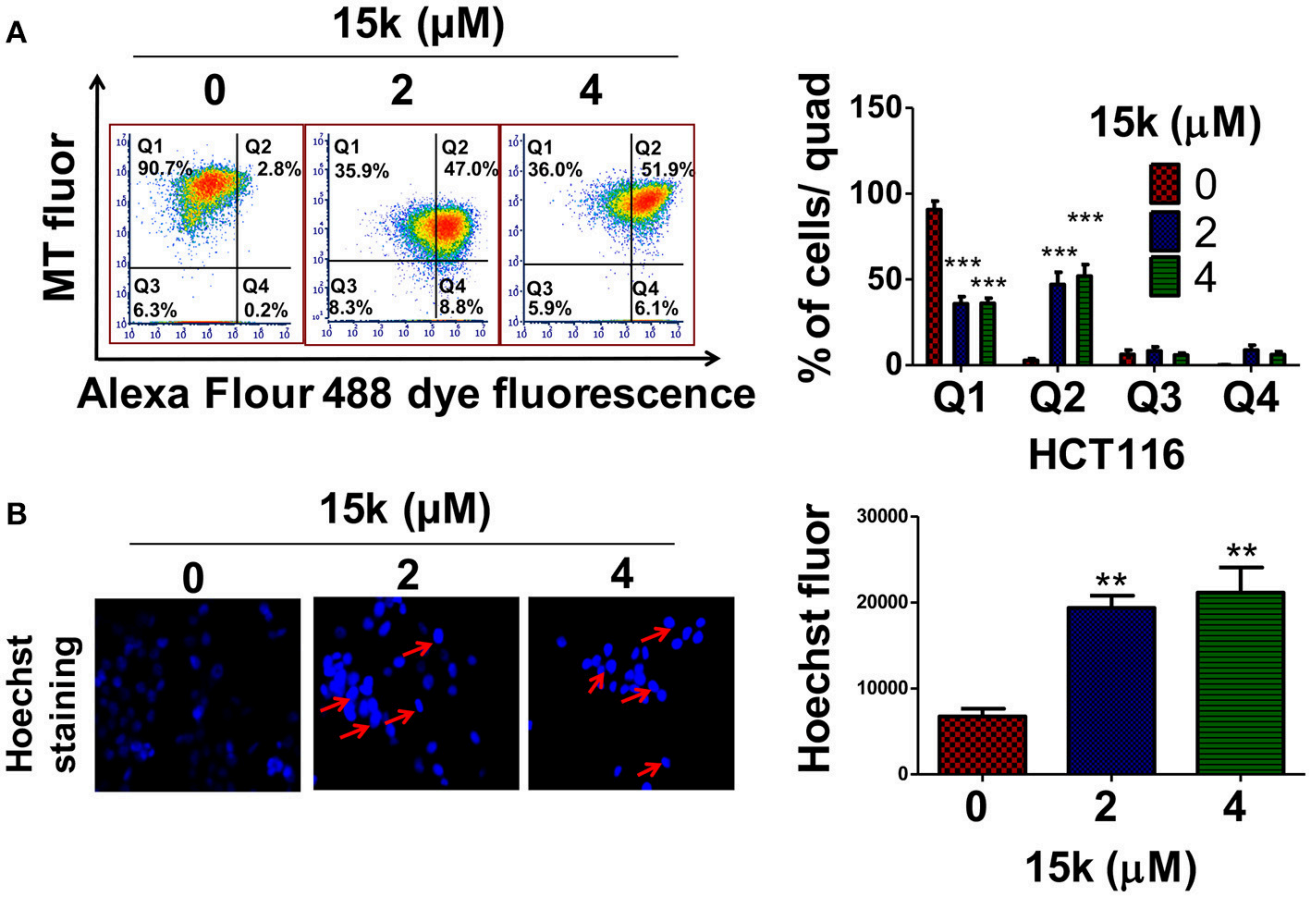
The effect of 15k on apoptosis at different concentrations in HCT116 cells; **(A)** HCT116 cells were incubated overnight with different concentrations (0, 2, or 4 μM) of 15k. Cells were then incubated with the reagents from the MitoTracker Red and Alexa Fluor 488 annexin V kits for flow cytometry. Representative results from three independent flow cytometry experiments are shown; a histogram summarizing the results for **(A)** is also shown; **(B)** the changes in the nuclear morphology of HCT116 upon overnight incubation with different concentrations (0, 2, 4 μM) of 15k are shown. The cells were fixed and stained with the DNA binding dye Hoechst 33342 dye. Condensed and fragmented nuclei were observed under an EVOS fluorescent microscope at 40X. A histogram summarizing the results is also shown; ^*^p < 0.05, ^**^p < 0.01, ^***^p < 0.001.

The inhibition of apoptosis is one of the major mechanisms involved in the pathogenesis and progression of different types of cancer (Wong, 2011). During apoptosis, the loss of mitochondrial membrane potential, which is related to the integrity of the inner mitochondrial membrane, is an early event in the apoptotic process that increases the mitochondrial membrane permeability, allowing for the release of apoptotic factors, including cytochrome c (Kroemer et al., 2007). We measured the mitochondrial membrane potential using the molecule Mitotraker red, which is concentrated in the mitochondria in a membrane potential—dependent manner (Kodiha et al., 2015). Also, fluorochrome-conjugated annexin V can be used to detect apoptosis based of the level of exposed phosphatidylserine (PS) on the cell membrane (Wlodkowic et al., 2012). Our results indicated that 90.66% of the vehicle incubated HCT116 cells were viable, with an intact mitochondrial membrane potential, as seen in quadrant I (Figure 3A) and only 2.83% showed apoptosis in quadrant II (Figure 3A). However, incubation with 2 or 4 μM of compound 15k produced a significant decrease in the number of viable cells in quadrant I (35.8 and 36.02%, respectively, Figure 3A, *p* < 0.001). This was associated with a significant increase in the percentage of HCT116 cells undergoing apoptosis and a reduction in their mitochondrial membrane potential with 2 or 4 μM (47 and 51%, respectively; *p* < 0.001 for both concentrations) in quadrant II (Figure 3A). Similarly, HCT116 cells incubated with 2 or 4 μM of compound 15j showed a significant shift (*p* < 0.001) from quadrant I in their fluorescence parameters. This indicates a significant reduction in membrane potential and increase in the number of cells undergoing apoptosis (*p* < 0.001 for 2 or 4 μM) in quadrant II compared to control cells (Data not shown).

Hoechst 33342 staining, as expected, in control (vehicle) HCT116 cells, revealed few to no apoptotic cells after incubation with vehicle (control) (Figure 3B). In contrast, 2 or 4 μM of compound 15k (*p* < 0.01) produced a significant increase in blue fluorescence, as well as highly condensed, fragmented nuclei chromatin (Figure 3B). 15j produced similar nuclear condensation (*p* < 0.05) (Data not shown). Due to the higher selectivity of compound 15k compared to 15j, we only conducted detailed mechanism of action experiments for compound 15k.

**Figure 4:**
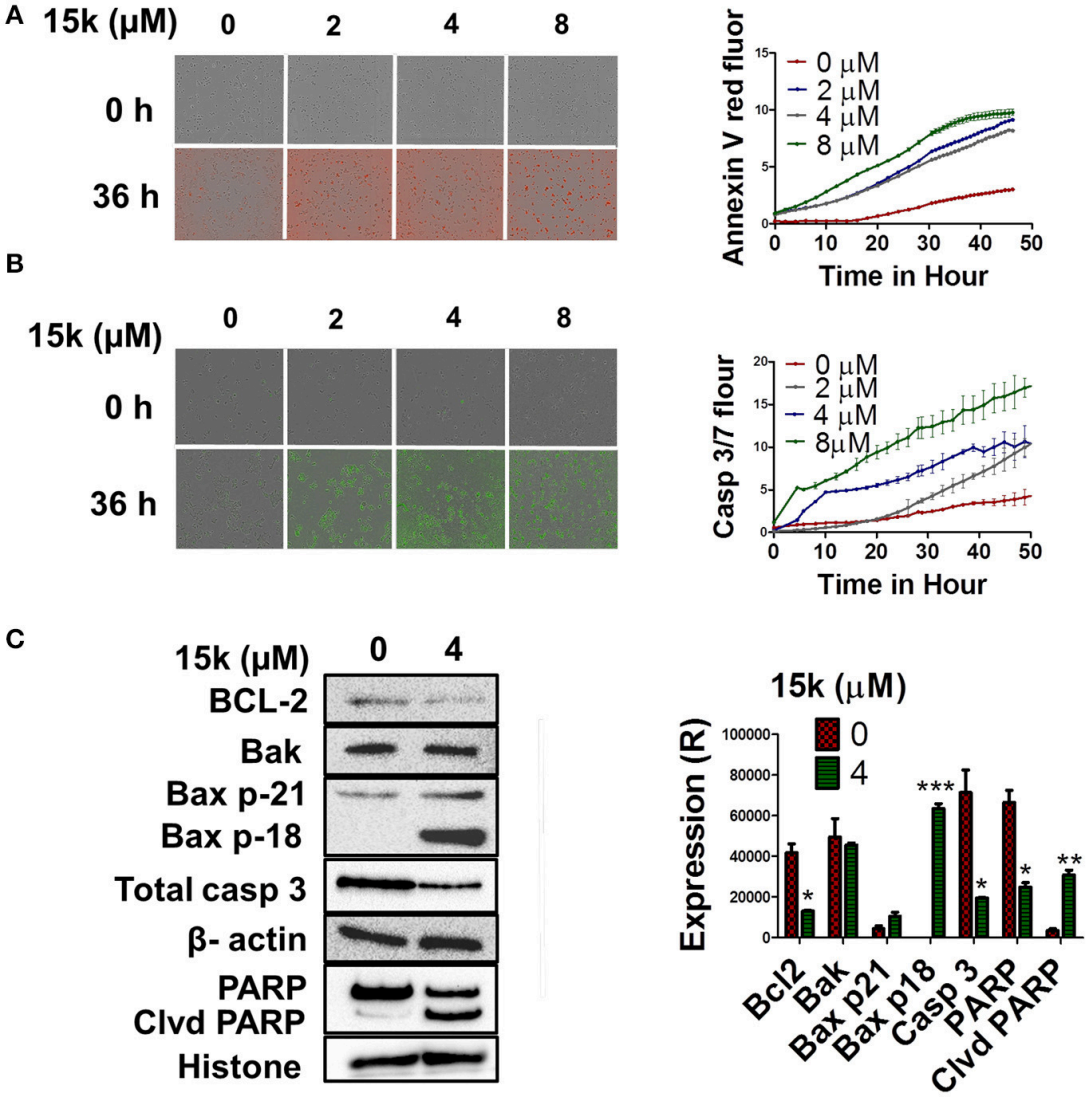
The effect of 15k on apoptosis induction in fluorescence time dependent studies at different concentrations in HCT116 cells. Apoptosis induction in fluorescence time dependent studies (Annexin V red, and caspase 3/7 reagent) on HCT116 cells incubated with different concentrations of 15k (0, 2, 4, and 8μM) for 48 h. The fluorescent reagents were added along with vehicle or 15k for 48 h. Annexin V (red fluorescence) and caspases 3/7 reagent (green fluorescence). **(A,B)** are representative pictures of the fluorescence level of the two reagents at the 0 and 36 h time points. Time line curves quantitatively summarizing the results are also shown. **(C)** Western blots for the proteins BCL-2, Bak, Bax (p21 and p18), caspase-3, PARP, and cleaved PARP following overnight incubation with 15k (4μM). The values of the cytosolic proteins were normalized to β-actin levels where nuclear proteins (PARP) are normalized to histone levels. A histogram summarizing the levels of each protein is also shown. All the data are presented as the means ± SEM of three independent studies with ^*^p < 0.05, ^**^p < 0.01, ^***^p < 0.001 vs. control group.

Similarly, real-time quantification of apoptosis indicated that 15k induces apoptosis at early time points (Figures 4A,B). Compound 15k (0, 2, 4, or 8 μM) significantly increased annexin V red fluorescence over time compared to control. A significant difference in fluorescence between cells incubated with vehicle or 15k appeared after 12 h of incubation, indicating a significant induction of apoptosis (Figure 4A). Similarly, 15k induced apoptosis in a time-dependent manner by activating of caspases 3 and 7 (Figure 4B). The lowest concentration (2μM) of compound 15k required a longer incubation time (≈24 h) to induce apoptosis compared to the 4 and 8 μM concentrations (≈10 h; Figure 4B). Overall, these results indicate that compound 15k induces apoptotic cell death at early time points.

#### The effect of compound 15k on the intrinsic apoptosis pathway

Apoptosis is primarily induced by the activation of the intrinsic and extrinsic pathways (Adams and Cory, 2007; Ashkenazi, 2008). The Bcl-2 family of proteins plays an integral role in the regulation of the intrinsic mitochondrial—dependent apoptotic pathway (Gross et al., 1999; Czabotar et al., 2014). It is well established that Bcl-2 is an antiapoptotic protein, whereas Bax and Bak are proapototic proteins that activate caspase 3, mediating the subsequent cleavage of cellular proteins, such as nuclear poly ADP ribose polymerase (PARP) (Leibowitz and Yu, 2010). To determine whether 15k is mediating its apoptotic effect through the induction of the intrinsic pathway, we analyzed the levels of the most important proteins in this pathway. Our Western blot data, obtained using HCT116 cells, indicated that compound 15k, at 4μM, significantly decreased the levels of Bcl-2 (*p* < 0.05, Figure 4C), but had no significant effect on Bak levels (Figure 4C). Interestingly, compound 15k, at 4μM, significantly (*p* < 0.001) induced the cleavage of the protein Bax (p 21), eliciting the formation of the more potent fragment of Bax, p18, which is absent in untreated cells (Figure 4C). These results suggest that compound 15k mediates its apoptotic effects primarily via the cleavage of Bax p21 to Bax p18 and by decreasing Bcl-2 protein levels.

We also investigated the effect of 15k on caspase 3 and its nuclear substrate, PARP. Our results showed that compound 15k induced the cleavage of total caspase in HCT116 cells incubated with 4 μM of 15k, compared to control cells (Figure 4C, *p* < 0.05). In addition, nuclear PARP was also cleaved significantly, indicating that compound 15k produces a significant activation of caspase 3. Compound 15k, at 4 μM, significantly reduced (*p* < 0.05) total PARP compared to untreated or control cells (*p* < 0.01, Figure 4C).

#### Effect of compound 15k on apoptosis in cells lacking Bax, Bak, and Bax and Bak proteins

**Figure 5:**
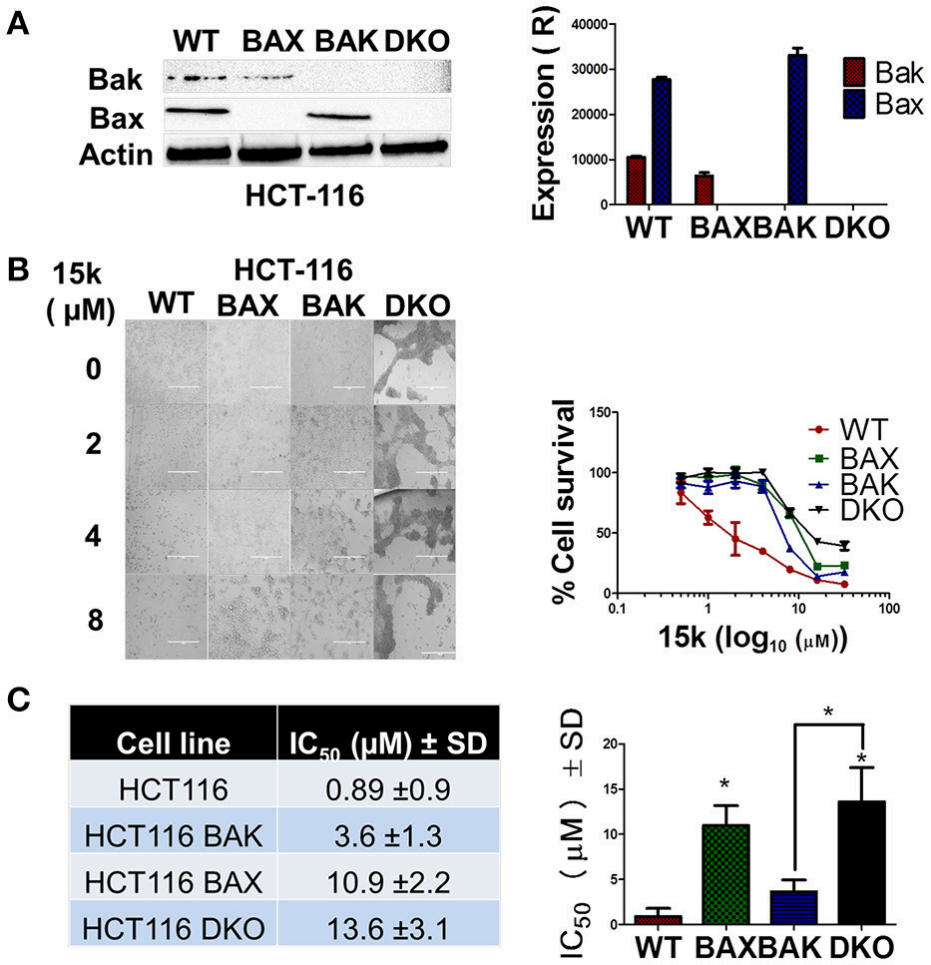
Wild type HCTT16 cells are more sensitive to 15k compared to knockout cells; **(A)** Western blot analysis showing the lack of expression of apoptotic proteins (Bak and Bax) in the HCT116 knock out cell lines (WT, Bak–/–, Bax–/–, and Bak-Bax–/–) to verify the knockout in the different cell lines. A bar graph summarizing the results is also shown; **(B)** Morphological analysis, using an EVOS microscope (at 20X) of the cytotoxic effects of 15k (0, 1, 5, and 10 μM) on HCT116 cell lines with different apoptotic genes knocked out 68 h after incubation. The cells were photographed for each triplicate treatment with an EVOS microscope. A survival curve of HCT116 knockout cells compared to wild type cells is also shown **(C)** IC_50_ Values of 15k for HCT116 knockout cells compared to wild type cells (WT). A bar graph comparing the IC_50_ values on different cells is also shown. Cell viability was determined using the MTT assay. IC_50_ values are represented as means ± SD of three independent experiments performed in triplicate with ^*^p < 0.05.

We conducted experiments to determine the cytotoxicity of compound 15k on different HCT116 cell lines that had the following genes knocked out: Bax–/–, Bak–/– and the Bax-Bak double knockout (DKO, Bax-Bak–/–), thereby abrogating the expression of the proteins Bax, Bak, and Bax and Bak, respectively, and compared its cytotoxicity in wild type (WT) HCT116 cells. Western blot experiments indicated the absence of Bax, Bak, and Bax and Bak in cells with genes knocked out for these proteins (Figure 5A). Next, we determined the cytotoxicity of compound 15k on WT, BAK, Bax, and DKO knockout cells. The HCT116 cells were the most sensitive to the apoptotic effects of compound 15k, with an IC_50_ value of 0.89 μM (Figures 5B,C). The Bak knockout cells had a higher IC_50_ value (3.6 μM), which was not significantly different from WT cells (Figures 5B,C). However, the Bax knockout cells were significantly less sensitive (*p* < 0.05) to compound 15k, with an IC_50_ value 10.9 μM. Finally, the DKO cells were the least sensitive of all the cell lines, with an IC_50_ value of 13.6 μM (*p* < 0.05 compared to HCT116 WT cells, Figures 5B,C). These results are consistent with the previous results indicating that the anticancer efficacy of compound 15k is due, in part, to altering the expression of intrinsic apoptotic signaling proteins, mainly Bax.

### 15k inhibits α-tubulin dynamics and expression in CRC cells

**Figure 6:**
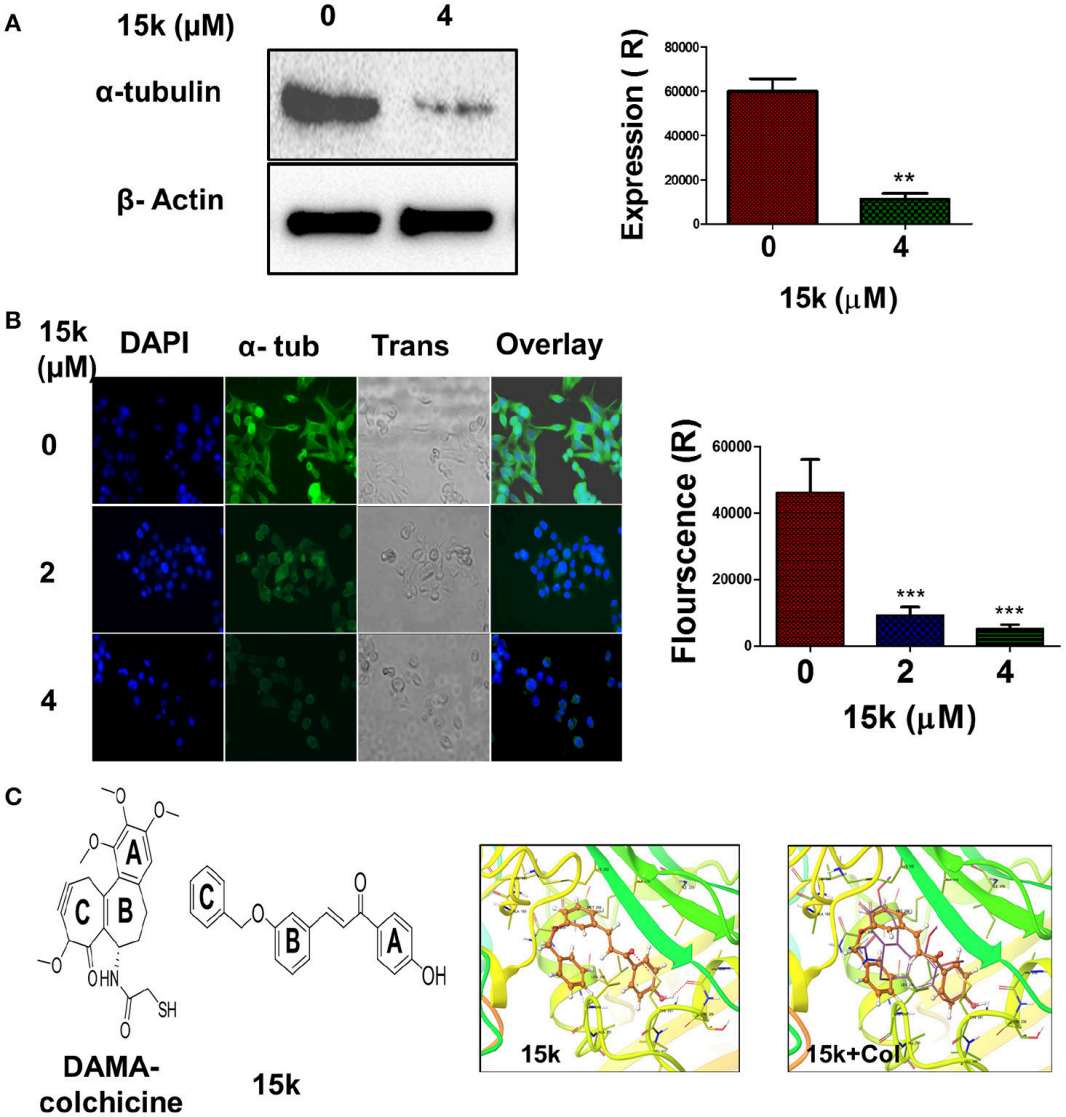
The effect of 15k on the expression of α- tubulin protein level in HCT116 cells; **(A)** Western blot showing the protein level of α-tubulin following overnight incubation with 15k (0, 4 uM). A histogram quantitatively summarizing the results of expression is also shown where Expression R stands for relative expression; **(B)** The effect of 15k on spindle microtubule formation in HCT116 cells. Representative pictures of α-tubulin fluorescence from cells incubated overnight with 15k at different concentrations (0, 2, 4 μM). Cells were then fixed and labeled with a monoclonal α-tubulin (green) antibody conjugated to FITC and nuclei stained with DAPI. A histogram quantitatively summarizing the results is also shown. The data are presented as the means ± SEM of three independent studies with ^**^p < 0.01, ^***^p < 0.001; **(C)** The docking studies with the structure of DAMA Colchicine (Col) and 15k, respectively, flowed by XP-Glide predicted binding mode of 15k in the colchicine binding site of tubulin (PDB ID: 1SA0). Important amino acids are depicted as sticks with the atoms colored as carbon—green, hydrogen—white, nitrogen—blue, oxygen—red, sulfur—yellow, whereas the ligand 15k is shown with the same color scheme as above except for carbon atoms which are represented in orange. The red dotted lines represent hydrogen bonding. An overlay of the ligand 15k on DAMA-colchicine (carbon atoms presented in yellow) in the colchicine binding site of tubulin is also shown.

The efficacy of 4 μM of 15k to inhibit α-tubulin in HCT116 cell lines was determined after overnight incubation. Compound 15k, at 4 μM, produced a significant decrease (*p* < 0.01) in the expression of α-tubulin in HCT116 cells compared to cells incubated with vehicle (Figure 6A). Immunofluorescence staining of the cells further confirmed the effect on the dynamics of cellular microtubules. The control HCT116 cells showed a significant green fluorescence under an EVOS microscope, with well-organized, spindle—like microtubules that were arrayed along the long axes in the cytoplasm around the nucleus (Figure 6B). The incubation of HCT116 cells with compound 15k (2 or 4 μM) produced a significant reduction (*p* < 0.001 for both concentrations) in the α-tubulin green fluorescence compared to the control cells (Figure 6B). Furthermore, the shape and integrity of the cellular microtubules were significantly disrupted and the cells were more rounded, with a loss of their spindle-like shape (Figure 6B).

Molecular docking studies were conducted to determine the mechanism by which microtubule assembly was inhibited by compound 15k. The colchicine binding site of tubulin (PDB ID: 1SA0, 3.58 Å) was used as a docking model as several antitubulin chalcones have been reported to bind to this site (Ducki, 2009; Lu Y. et al., 2012). The docking of the ligands to the colchicine binding site of tubulin was performed using the Glide (Grid-Based Ligand Docking with Energetics) program of Schrödinger molecular modeling suite (Schrödinger, Inc., New York, NY, 2012) in extra precision mode (XP). The validation of the docking protocol was carried out by redocking DAMA-colchicine extracted from the X-ray structure and evaluating the similarity of docked conformation to observed X-ray crystallographic conformation in terms of root mean square deviation (rmsd). The low rmsd value was <0.69, indicating the applicability of the docking protocol for the present studies. Subsequently, the conformational library generated for compound 15k was docked at the colchicine binding site of tubulin and the best fit model was selected on the basis of Glide score and visual inspection. Figure 6C depicts the XP glide predicted docked model of 15k in the colchicine-binding site of tubulin. In this model, the chalcone moiety was found to align with rings A and C of colchicine in the colchicine-binding site of tubulin. This alignment places the phenyl ring B of chalcone over ring A of colchicine, forming hydrophobic contacts with Lys352β, Val181α, Asn258β and Met259β and Ala316β, whereas the phenyl ring A of the chalcone moiety is stabilized in the binding cavity through hydrophobic interactions with Ile378β, Val238β, Thr239β, Leu242β, Ala250β, and Leu255β. This positioning of the chalcone scaffold enables hydrogen bonding interaction between the carbonyl oxygen of enone group in chalcone moiety and sulfhydryl group of Cys241β. This interaction is significant because colchicine shows a similar interaction with tubulin. Another hydrogen bond was present between the phenolic “OH” in para position phenyl ring B and nitrogen of Leu255β. These two hydrogen bonds play a crucial role in stabilizing the conformation of 15k at the colchicine binding site. The unsaturated intermediate chain between rings A and B of chalcone moiety is also positioned is placed in close vicinity of the side-chains of Ala316β and Leu255β. The phenyl ring C of 15k forms a “cis-like” configuration relative to ring B of the chalcone moiety, establishing hydrophobic contacts with Ala250β, Lys254β, and Leu255β. All of above mentioned interactions explain the highly significant inhibition of tubulin observed with compound 15k.

### 15k reverses metastasis in CRC cells

**Figure 7:**
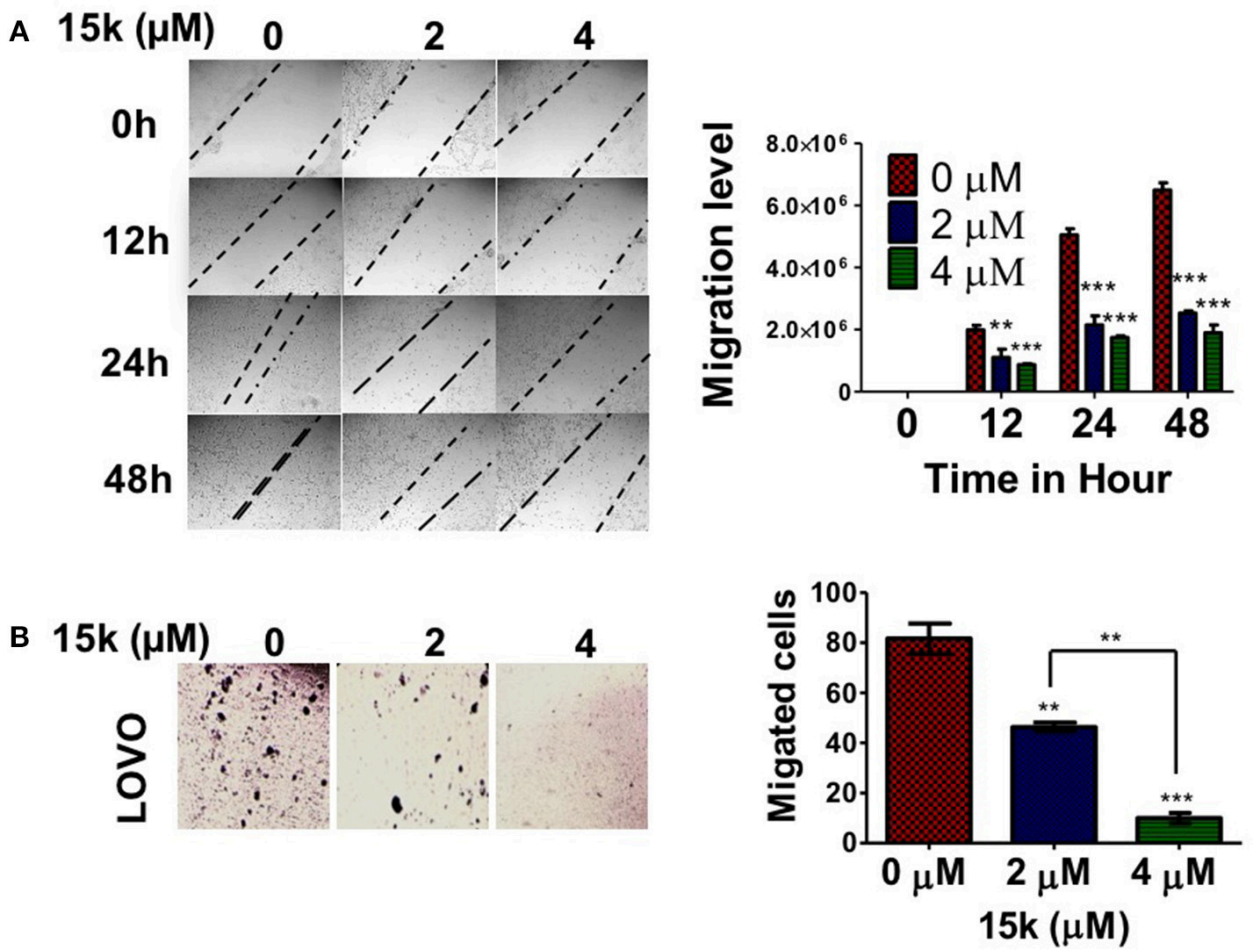
The effect of 15k on CRC (LOVO) cell migration and invasiveness. For the wound healing assay, the cells were wounded and incubated with 0, 2, or 4 μM of 15k for 48 h; **(A)** representative images showing LOVO cell migration. The pictures were taken at time points 0, 12, 24 and 48 h. A bar graph showing the quantitative analysis of a wound healing assay. For the transwell migration assay, the cells were seeded on a membrane with 8 μm pore size and incubated for 24 h with different concentrations of 15k; **(B)** representative images showing the number of LOVO cells that were able to cross the membrane (representing invasiveness) after 24 h of incubation. The cells were counted/field of image and the data are presented as a bar graph. All data are presented as the means ± SEM of three independent experiments with ^**^p < 0.01, ^***^p < 0.001.

The development of cancer metastasis is dependent on the migration and invasive properties of cancerous cells (van Zijl et al., 2011). The wound healing and transwell migration assays were used with LOVO cells, as a model for metastatic CRC as it is characterized by a high level of invasiveness (Fan et al., 2015). The wound healing assay is a well-established method to detect and analyze cell migration potential (Liang et al., 2007). LOVO cells incubated with compound 15k migrated significantly slower than vehicle-incubated LOVO cells at 24 h (*p* < 0.05 for 2 μM and *p* < 0.01 for 4μM, Figure 7A) and 48 h (*p* <0.01 for 2μM and *p* < 0.001 for 4 μM, Figure 7A). Control cells showed a complete closure of the wound after 48 h. In the transwell migration assay, another standard *in vitro* assay to assess cell invasiveness (Albini and Benelli, 2007),15k significantly decreased the number of LOVO cells that migrated through the transwell polycarbonate filters compared to the vehicle-incubated cells (*p* < 0.01 μM for 2μM and <0.001 for 4μM after 24 h, Figure 7B). Thus, based on these results, compound 15k decreases the metastatic potential of CRC cells lines *in vitro* by decreasing their migratory activity. The effect of compound 15k on the invasiveness of COLO 205 cell line was also measured using trans-well migration assay. 15k significantly inhibited the migration of COLO 205 cell (*p* < 0.01 for both 2 and 4 μM, data not shown). 15j also reversed cell migration and metastasis at different concentrations. LOVO cells incubated with 15j migrated significantly slower than control cells at 24 h (*p* < 0.05 for 2 μM and *p* < 0.01 for 4 μM) and 48 h (*p* < 0.01 for 2 μM and *p* < 0.001 for 4 μM, data not shown). 15j also significantly reduced the trans-well migration of LOVO (*p* < 0.001 for both 2 and 4 μM) and COLO 205 cells (*p* < 0.05 for 2 μM and *p* < 0.01 for 4 μM, data not shown).

### Compound 15k significantly inhibits the Wnt/β-catenin/EMT pathway in CRC cells

**Figure 8:**
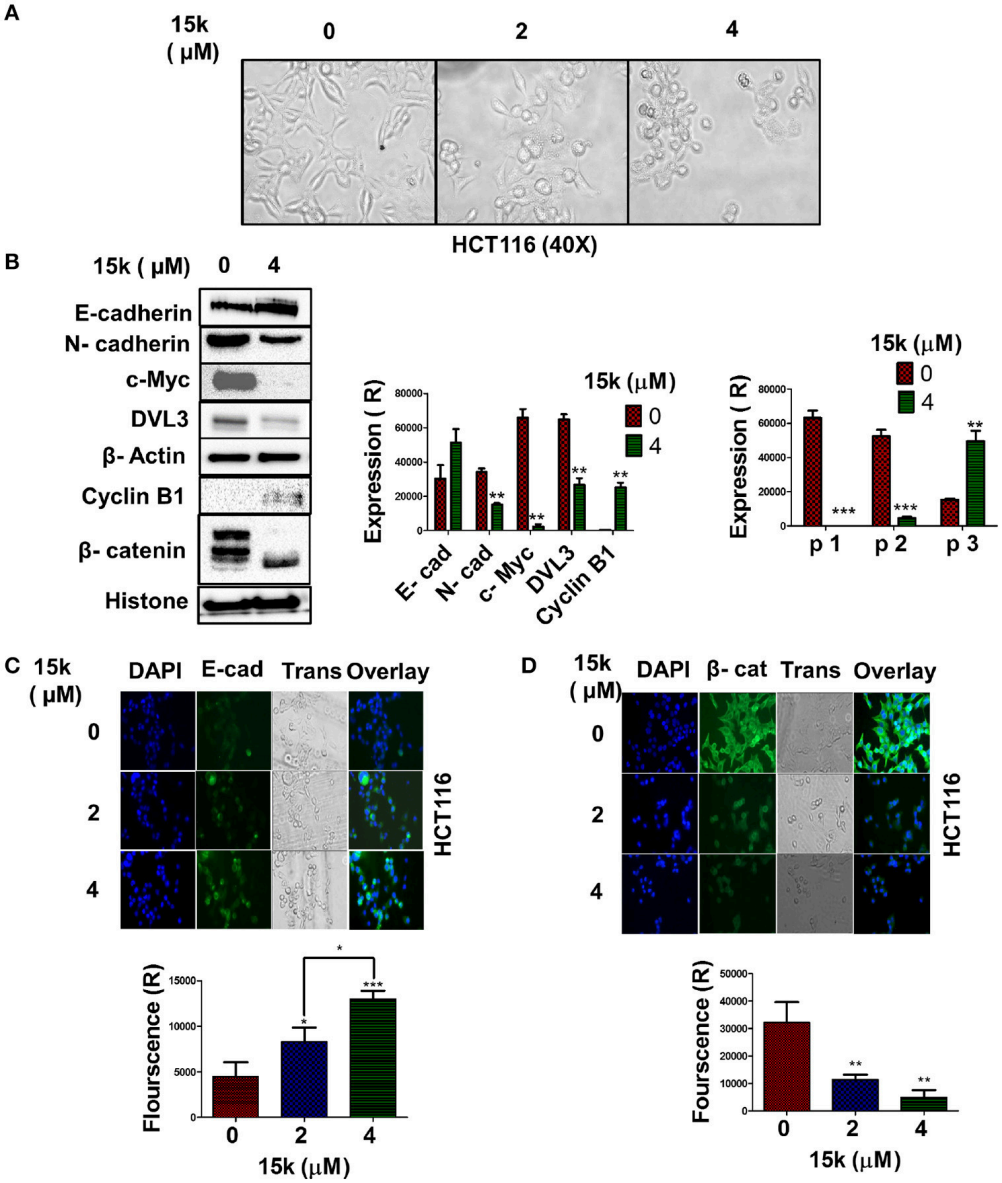
The effect of 15k on embryonic signaling pathways: **(A)** Representative image of HCT116 cell morphology at 40X. The morphological changes are consistent with EMT inhibition as the cells where converted from a mesenchymal shape to epithelial shape. Images were taken from EVOS microscope; **(B)** Western blots illustrating the levels of E-cadherin, N-cadherin, DVL3, c-Myc, Cyclin B1, and β-catenin fragments following overnight incubation with 15k (4 μM). The values of the cytosolic proteins were normalized to β-actin and histone levels. A histogram quantitatively summarizing the results for each protein where expression (R) stands for the relative expression level of each protein and a histogram quantitatively summarizing the effects of 15k on the levels of the β-catenin protein fragments are both shown where P1, P2, and P3 are the fragments of β-catenin; **(C,D)** representative pictures of E-cadherin and β- catenin fluorescence in HCT116 cells incubated with 15k at 0, 2 or 4 μM for 24 h, respectively. Histograms quantitatively summarizing the results are shown (Fluorescence R is for relative fluorescence levels). All data are presented as the means ± SEM of three independent experiments with ^*^p < 0.05, ^**^p < 0.01, ^***^p < 0.001.

The aberrant, over-activation of embryonic signaling pathways, including Wnt/β-catenin/epithelial-mesenchymal transition (EMT), is involved in the development, progression, metastasis, and resistance of CRC (Du and Shim, 2016). Given that compound 15k significantly inhibited LOVO cells migration and invasiveness, we hypothesized that it may have repressive effects on the EMT pathway. To test this hypothesis, the morphology of HCT116 cell was observed following incubation with 15k. The results indicated that 15k significantly changed the morphology of the cells from mesenchymal to a more epithelial-like appearance (Figure 8A). We then determined the efficacy of compound 15k on the expression of (1) β-catenin; (2) E-cadherin, and (3) N-cadherin in CRC cells. Compound 15k (4 μM) significantly downregulated the level of N-cadherin (*p* < 0.01) compared to control cells (Figure 8B). However, E-cadherin expression was not significantly altered by compound 15k, (Figure 8B). For nuclear β-catenin in HCT116 cells, 15k induced the cleavage of the full-length protein (p1 and p2) to lower molecular weight fragments (Close to 70 kDa, p3), thereby significantly lowering its level in cells incubated with 15k compared to control cells (*p* < 0.001 for p1 and p2, Figure 8B). The proteolytic fragmentation of β-catenin is probably induced by apoptosis as fragment p3 was only detected at a significant level at HCT116 cells incubated with compound 15k (*p* < 0.01 compared to cells incubated with vehicle, Figure 8B). These results were further confirmed by immunostaining with E-cadherin and β-catenin fluorescent antibodies after incubating cells with 15k (0, 2, and 4 μM). Indeed, E-cadherin expression was significantly increased in HCT116 cells incubated with 15k (*p* < 0.05 for 2 μM, *p* < 0.001 for 4 μM, Figures 8C,D) and there was a significant decrease in the level of β-catenin available for nuclear translocation (*p* < 0.01 for 2 and 4 μM of compound 15k compared to cells incubated with vehicle, Figures 8C,D).

Disheveled protein 3 (DVL3) is another protein involved in the Wnt/β-catenin signaling pathway (MacDonald et al., 2009). 15k, at 4 μM, significantly decreased the levels of DVL3 compared to cells incubated with vehicle (*p* < 0.01, Figure 8B). The myc gene (c-Myc) codes for a protein that mediates the transformation of the epithelial phenotype into a mesenchymal phenotype during carcinogenesis (Cho et al., 2010). Also, c-Myc inhibits the activity of the protein GSK3β, which catalyzes the phosphorylation and subsequent inactivation of β-catenin (Cho et al., 2010). The incubation of cells with compound 15k (4 μM) produced a significant decrease in the levels of c-Myc protein compared to control cells (*p* < 0.01, Figure 8B). Data suggest that cyclin B1 is involved in the development and progression of cancer (Androic et al., 2008). However, a recent study showed that cyclin B1 overexpression is negatively correlated with the development of metastasis in CRC (Fang et al., 2015). Indeed, the inhibition of cyclin B1 expression was found to increase the invasive potential of CRC cells *in vitro* (Fang et al., 2015). In addition, the inhibition of cyclin B1 significantly induced EMT and the subsequent loss of E-cadherin expression (Fang et al., 2015). Therefore, we determined the effect of compound 15k on the expression of cyclin B1 levels using Western blotting. As previously reported (Fang et al., 2015), control HCT116 cells expressed low levels of cyclin B1 (Figure 8B). However, 4 μM of compound 15k induced a significant increase in the expression levels of cyclin B1 (*p* < 0.01, Figure 8B). These results suggest that the inhibition of the expression of cyclin B1 may contribute to its efficacy.

## Discussion

In this study, novel silybin derivatives were screened for cytotoxicity in CRC cells. Compound 15k had significant anticancer efficacy, with IC_50_ values ranging from 1 to 2μM for HCT116, S1, and LOVO cell lines. Data indicate that silybin (the parent compound of 15k) has significant efficacy against different types of cancer, including CRC. The proposed mechanisms for silybin's anticancer efficacy include induction of apoptosis and inhibition of Wnt/β-catenin/EMT pathways. For example, previous studies have reported that silybin inhibits the proliferation of the CRC lines HCT116 and HT29 at 50–200μM (Agarwal et al., 2003). Furthermore, silybin induces both G1 and G2 cell cycle arrest and induces apoptosis (Agarwal et al., 2003). Caspase 3 activation and cleavage of PARP occur in cancer cells incubated with silybin (Agarwal et al., 2003; Kaur et al., 2010). In this study, the silybin derivative, 15k, also induced cytotoxicity in CRC cells (including LOVO), cell cycle arrest in the S/G2 phases, ROS generation and apoptosis through the intrinsic apoptotic pathway and activated caspase 3 at significantly lower concentrations (1–2μM) than silybin.

Our results indicated that 4μM of compound 15k induced the cleavage of Bax p21 into Bax p18. This 15k-indcued cleavage maybe, in part, responsible for the significant cytotoxicity of 15k on HCT116 cells. The cleavage of the pro-apoptotic protein, Bax, has been reported to enhance its apoptotic and cell death efficacy. The cleavage of Bax p21 to its more active p18 fragment was detected in B-cell chronic lymphocytic leukemia (B-CLL) incubated with camptothecin analog, 9-amino-20(s)-camptothecin, or the purine analog, fludarabine (Thomas et al., 1996). The same results were also reported = in hematopoietic leukemia cell lines HL-60 undergoing apoptosis (Wood and Newcomb, 1999). When the efficacy of Bax p21 and p18 were compared on the induction of cell death in human embryonic kidney (HEK 293 T), p18 was significantly more cytotoxic (Wood and Newcomb, 2000). The caspase inhibitor, Z-VAD-fmk, completely inhibited the activity of p21, but only partially blocked p18 efficacy (Wood and Newcomb, 2000).

The antimetastatic effect of 15k was also demonstrated in LOVO cell line as it significantly reduced the motility and invasiveness of these cells *in vitro*. Previously, it has been reported that silybin has anti-metastatic efficacy (Ramasamy and Agarwal, 2008). For example, wound healing and trans-well migration assays conducted with PC3 and PC3MM2 cells, incubated with silybin, indicate that silybin, at 30 and 90 μM, inhibits the migratory and invasive capacities of these cells (Deep et al., 2011). Silybin, at 100 and 200 μM, also inhibits the invasiveness of ARCaP(M) cells and suppresses the potential of these cells to metastasize to bone (Wu et al., 2009). We showed that compound 15k produced similar anti-metastatic effects *in vitro* in metastatic LOVO CRC cell lines, as it significantly inhibited wound closure and suppressed the transwell migration levels in LOVO cells.

Compound 15k significantly (1) downregulated the EMT marker and inducers N- cadherin, β- catenin, c-Myc, and DVL3 and (2) upregulated epithelial marker E- cadherin. Silybin, at 0.5 and 1% (w/w), has been reported to significantly upregulate E-cadherin and decrease the expression of the EMT markers, vimentin and MMP-2, in transgenic mice with prostate adenocarcinomas (Singh et al., 2008). A significant inhibition of EMT by silybin was also reported *in vitro*, in addition to inhibiting invasiveness in the metastatic prostate cancer cell lines, ARCaPM and DU145, which overexpress mesenchymal biomarkers and have a high potential to produce bone metastasis (Wu et al., 2009). Furthermore, the morphology of the cells was changed to epithelial—like cells. A significant upregulation in E-cadherin and downregulation in nuclear β-catenin levels were also reported in PC3 and PC3MM2 cells incubated with 90 μM of silybin (Deep et al., 2011). Furthermore, EMT—driven resistance was significantly decreased by silybin (100 mg/kg) via reducing the expression of miRNA-21, which induces EMT (Cufí et al., 2013). Similarly, N-cadherin levels were significantly reduced, in tandem with an upregulation of the epithelial marker E-cadherin by silybin, (100 mg/kg), in addition to erlotinib treatment (Cufí et al., 2013). Wnt/β-catenin signaling in the prostate cancer cells PC-3 and DU-145 and breast cancer cells MDAMB-231 and T-47D cells was significantly decreased by 100–200 μM of silybin (Lu W. et al., 2012). Here, we report that the novel silybin derivative, compound 15k, significantly inhibited the expression of N-cadherin and upregulated the levels E- cadherin at relatively low concentrations (2–4 μM), compared to silybin (100–200 uM). Also, compound 15k reversed the spindle—like shape of mesenchymal cells into more rounded, epithelial—like shapes.

In CRC cells, 50–200 μM of silybin significantly inhibited the expression of nuclear β-catenin, c-Myc and induced apoptosis in SW480 cells with a mutant APC gene (Kaur et al., 2010). The i.p administration of silybin (100 or 200 mg/kg) also produced similar effects in a xenograft model in mice using SW480 cells (Kaur et al., 2010). The protein GSK3β significantly inhibits the expression of nuclear β-catenin in HCT116 cells by downregulating β-catenin (Eo et al., 2016). In addition, the transcriptional efficacy of β-catenin is decreased by caspase 3 and proteasomal degradation. During apoptosis, caspase 3 initiates the cleavage of several proteins, including β-catenin, which produces lower molecular weight fragments (Steinhusen et al., 2000). Here, we report that compound15k downregulated the nuclear levels of full length, active β-catenin (p1 and p2) and induced its degradation to lower molecular fragments (p3), which most likely have less efficacy as transcription factors. This effect of 15k could contribute to its anticancer efficacy.

With a molecular weight of 482.44 g/mol, silybin has a multi-ring structure and poor miscibility with oil. These properties are associated with extensive metabolism, mainly glucuronidation and sulfation of its phenolic groups (7, 20) (Theodosiou et al., 2014) and low oral absorption. These aforementioned issues contribute to silybin's poor bioavailability, as well as its limited potency and efficacy (Dixit et al., 2007; Zhu et al., 2013; Amawi et al., 2017b). The silybin derivatives reported in our study were of lower molecular weight (i.e., 330.38) and had fewer multi-ring structures compared to silybin. The *in-silico* prediction of the pharmacokinetics of 15k compared to silybin indicate that 15k has more suitable PK properties, including improved oral absorption, less metabolic reactivity and improved Caco 2 and MDCK cell permeability (data not shown). However, the current study was limited to *in vitro* cell culture and future *in vivo* pharmacokinetic studies should be done to determine the absorption, metabolism, distribution, and elimination parameters of 15k.

In conclusion, novel silybin derivatives were screened for cytotoxicity in CRC cell lines. Overall, compound 15k was the most efficacious compound and had significantly greater selectivity in killing cancer cells compared to normal cells. 15k induced cytotoxicity (IC_50_ values in the MTT assay of 1–2 μM), induced cell cycle arrest at G2 and S phases and increased oxidative stress. The apoptotic efficacy of 15k resulted from the inhibition of bcl-2 and the induction of the cleavage of Bax, p21 to Bax, p18 as well as cleavage of caspase 3 and nuclear PARP proteins. Compound 15k was also efficacious in reversing metastasis and invasiveness of LOVO cell lines. It significantly altered the expression of several proteins involved in the embryonic signaling pathways (i.e., Wnt/β-catenin/ EMT). Notably, 15k significantly (1) upregulated the expression of E-cadherin and (2) downregulated N-cadherin, β-catenin, DVL3, and c-Myc. Compound 15k also significantly upregulated cyclin B1 expression, which could contribute to its anti-metastatic efficacy. Finally, additional studies are essential to determine if 15k is efficacious *in vivo*.

## Author contributions

HA and AT conceptualized the idea and designed the experiments. HA, NH, RA, LS, CK, and EM run the experiments. RR, PT, and KE helped in data analysis and designs of section of experiments; HA, CA, and AT wrote the paper. AT, CA, RR, KE, and PT proofed and intellectually contributed during the revision of the manuscript. AT supervised the entire study. All authors read and approved the final manuscript.

### Conflict of interest statement

The authors declare that the research was conducted in the absence of any commercial or financial relationships that could be construed as a potential conflict of interest.
